# Influence of Teacher-Student Relationships and Special Educational Needs on Student Engagement and Disengagement: A Correlational study

**DOI:** 10.3389/fpsyg.2021.708157

**Published:** 2021-07-14

**Authors:** Claudia P. Pérez-Salas, Victoria Parra, Fabiola Sáez-Delgado, Himmbler Olivares

**Affiliations:** ^1^Departamento de Psicología, Facultad de Ciencias Sociales, Universidad de Concepción, Concepción, Chile; ^2^Departamento de Infancia y Educación Básica, Facultad de Educación, Universidad Católica de Temuco, Temuco, Chile; ^3^Centro de Investigación en Educación y Desarrollo, Facultad de Educación, Universidad Católica de la Santísima Concepción, Concepción, Chile

**Keywords:** student engagement, student disengagement, special educational needs, teacher-student relationships, school participation

## Abstract

Contemporary educational research has found that student engagement and disengagement have a relevant influence on learning outcomes. However, research on the influence of teacher–student relationships in the engagement of students with special educational needs (SEN) is scarce. The purpose of this study is to analyze the impact of teacher–student relationships, peer support at school, family support for learning, opportunities to participate at school, and SEN on engagement and disengagement of students using a sample of secondary students with SEN and typical development (TD). Through a non-experimental, correlational, and cross-sectional design, we evaluated 1,020 high school students (340 with SEN and 680 with TD) in the 9th grade (13–19 years old, *M* = 14.8; *SD* = 0.89). Teacher–student relationships, peer support at school, and family support for learning were assessed *via* subscales from the Student Engagement Inventory (SEI), opportunities to participate at school were measured with a subscale of the School Participation Questionnaire (SP), whereas engagement and disengagement were measured using the Multidimensional Scale of School Engagement (MSSE). Results show significant statistical differences between SEN and TD students in both student engagement and disengagement indicators. Engagement of SEN students is higher in the cognitive, emotional, and social dimensions than that of TD students. However, they also have higher disengagement in the cognitive and behavioral dimensions. Furthermore, SEN students rate their relationships with teachers more highly and perceive more opportunities for school participation than their peers. Further analyses show that teacher–student relationships are positively associated with all dimensions of student engagement and inversely with behavioral and cognitive disengagement. Although correlational, the findings suggest teacher–student relationships and school participation opportunities could be important variables for diminishing disengagement and its negative consequences for both SEN and TD students, while improving student engagement. We discuss these results considering possible implications for educational policies, practices, and research.

## Introduction

Student engagement is the quality of involvement of students with school activities (Skinner and Pitzer, 2012) including their participation in learning activities and interactions with teachers and peers. As a theoretical construct, student engagement is a multidimensional concept that involves distinctive and interrelated dimensions, such as student behaviors, emotions, and cognitive beliefs about school and learning (Fredricks et al., 2004). Behavioral engagement involves attendance and participation in academic and extracurricular activities. Emotional engagement involves positive and negative reactions to school, teachers, and peers (Finn, 1989; Voelkl, 1997), and cognitive engagement refers to the effort invested in learning (Fredricks et al., 2004). Recently, social engagement has been added as a dimension and refers to the quality of social interactions of students in the context of classroom tasks and the broader school context (Linnenbrink-Garcia et al., 2011; Rimm-Kaufman et al., 2015; Wang et al., 2016).

There is a vast literature on student engagement and its relationship with academic achievement (Fredricks et al., 2004; Chang et al., 2016): higher attendance rates, lower dropout rates, and fewer antisocial behaviors among pre-school, primary, and secondary students (Fredricks et al., 2004; Wigfield et al., 2006; Wang and Holcombe, 2010; Shin and Ryan, 2012).

Student engagement is understood as part of a broader motivational process with the learning context feeding back the conceptualization of individuals of themselves (Fredricks et al., 2019). As the self-system model states (Connell and Wellborn, 1991), individual and contextual factors influence student engagement based on how the school context helps to satisfy three relevant needs for the individual: relatedness, autonomy, and competence. The need for relatedness refers to the way in which the individual feels safe, connected, and valued by others. Autonomy is related to the need to experience agency over own behavior of an individual, both in its initiation and regulation and in the maintenance of the activity. Competence is related to the degree to which the individual knows how to obtain certain positive results and avoid negative ones. When psychological needs are met, engagement occurs, which manifests in emotion, cognition, and behavior. However, when these psychological needs are not satisfied, disaffection with the school will arise (Connell and Wellborn, 1991).

School disengagement relates to maladaptive behaviors and attitudes toward schools and learning, and it reflects the ways in which students begin to withdraw and become disaffected with school (Skinner et al., 2008). It has been associated with negative outcomes, including low achievement, disruptive and risky behaviors, and psychological problems (Morrison et al., 2002; Wang and Fredricks, 2014). Disengagement is a multidimensional construct that involves the behavioral, emotional, cognitive (Skinner et al., 2008; Wang et al., 2017), and social dimensions (Wang et al., 2017). Wang et al. (2017) specify that behavioral disengagement includes getting in trouble at school, not paying attention in class or goofing off, and finding ways to be late for school or getting out of classes. Cognitive disengagement involves giving up quickly and speeding through homework rather than trying to understand or benefit from it. Emotional disengagement is feeling worried, overwhelmed, and frustrated in school. Finally, social disengagement implies a student feels invisible at school and does not consider interaction with others an important aspect of his school life.

Initially, researchers treated engagement and disengagement as opposite poles of the same continuum. However, this approach disregards the fact that disengagement is more than the absence of engagement, but the presence of maladaptive processes (Skinner et al., 2009). Engagement and disengagement are not fixed states, and student levels of both constructs vary over time (Jang et al., 2016; Burns et al., 2019). In the secondary school years, engagement tends to decrease (Burns et al., 2018; Engels et al., 2021) and disengagement increases (Burns et al., 2019; Engels et al., 2021). Hence, although engagement and disengagement are related constructs, measuring them separately can potentially provide more nuanced information regarding the phenomena, as disengagement captures aspects that engagement cannot (Jang et al., 2016; Bergdahl et al., 2020).

Unfortunately, most studies on the engagement and disengagement of students have focused on students with typical development (TD) (O'Donnell and Reschly, 2020). Consequently, little is known about the engagement or disengagement of students with special educational needs (SEN), especially those enrolled at mainstream schools (Schindler, 2018). This is, however, starting to change because of the importance of engagement and disengagement in academic achievement (Moreira et al., 2015). Studying the student engagement of SEN students is important since these students face significant challenges in school, and there is building evidence on the academic, social, and psychological consequences of their school struggles (Douglas et al., 2012; Cortiella and Horowitz, 2014; Moreira et al., 2015). However, as Moreira et al. (2015) reported, studies providing this evidence are not conclusive and present mixed results. Some found lower levels of engagement for SEN students compared with their TD peers, whereas others showed no differences in engagement between the two groups.

Comparisons of engagement between SEN and TD students in the context of inclusive settings have also yielded inconclusive results. Employing an eco-behavioral observation tool with adolescents in inclusive classrooms, Wallace et al. (2002) found no differences in academic and behavioral engagement. Both groups showed high levels of academic engagement and low levels of inappropriate behaviors. Furthermore, using large-scale survey data (*N* = 10,000) of 5–9th-grade pupils, Schindler (2018) obtained lower scores in all dimensions of engagement for SEN students (motivation and effort, belonging/well-being at school, participation in learning activities, and participation in social activities). The raw difference was larger for motivation and effort: SEN students scored a.7 SD lower than TD students. According to Schindler (2018), the differences in engagement between SEN and TD students in her research cannot be explained by differences in backgrounds of students or at the school level. Yang et al. (2020), in a research project with 118 secondary school students with special needs integrated into mainstream schools, reported intermediate levels of student engagement (*M* = 3.10; *SD* = 0.85) on the five-point Likert School Engagement Scale of Fredricks et al. (2005).

The inconclusive results on the student engagement of SEN students can be attributed to conceptual and methodological reasons. First, different studies conceptualize student engagement in different ways (unidimensional/multidimensional), the definition of engagement dimensions differ (e.g., including social or academic dimensions besides cognitive/behavioral/emotional-affective or measuring only one of them) (Moreira et al., 2015; O'Donnell and Reschly, 2020), and variation in terms of whether engagement is measured on a single continuum (low or high) or there is a separate measurement of engagement and disengagement (O'Donnell and Reschly, 2020). Douglas et al. (2012) state that most studies on the engagement of SEN students use either behavioral (e.g., attendance, dropouts, and participation in school activities) or cognitive indicators of engagement (e.g., achievement in specific subjects, such as math or literacy), and disregard the emotional and social aspects thereof. These elements highlight the need for more research in this field considering all dimensions involved in student engagement.

Age could also be an important variable when studying these concepts. For example, Janosz et al. (2008) found different types of engagement trajectories for 12–16-year-old students. One of these pathways (2% of the overall sample) contained around one-third of the SEN students (the most common for those students). It characterized a decreasing pattern of engagement. That is, these adolescents reported very high levels of school engagement at age 12, which rapidly decreased to the lowest levels in the study by age 16. Although not all students in the “decreasing pattern of engagement” trajectory had SEN, researchers should keep this finding in mind when comparing engagement of SEN and TD students because the results could be age dependent.

Regarding the variables involved in student engagement, Fredricks et al. (2004) describe three main groups: school-level factors (e.g., school size and opportunities for participating), classroom context (e.g., teacher–student relationships, peer acceptance, and classroom structure), and individual needs (e.g., relatedness, autonomy, and competence). Among these factors, the quality of teacher–student relationships has been identified as a key element in engagement and disengagement, including cognitive, behavioral, and emotional components for TD students (e.g., Roorda et al., 2011, 2017; Quin, 2017). Research showed that positive teacher–student relationships in high school contribute to adaptive behaviors and improve intentions to graduate (Burns et al., 2019; Burns, 2020). Furthermore, the perception of students of high levels of emotional and instructional support from teachers has been positively associated with emotional and behavioral engagement (Skinner et al., 2008; Havik and Westergård, 2020). Martin and Collie (2019) found that positive relationships of high school students with their teachers predict greater school engagement, and importantly, engagement is higher as the number of positive relationships outnumbered negative ones.

The association between engagement and teacher–student relationships has been studied through several paradigms: From the *self-system model perspective*, the quality of interacting with teachers provides information to adolescents that they are competent to succeed at school, related to others in these settings, and are autonomous learners (e.g., Roorda et al., 2011; Wang and Eccles, 2013; Krane et al., 2016). *Attachment theory* states that teachers who create warm, safe, and supportive relationships with their students can serve as important non-parental attachment figures and role models (Bergin and Bergin, 2009). Thus, students could use teachers as a safe base from which to explore the environment and engage in learning activities knowing they have support even in stressful situations (Verschueren and Koomen, 2012). Affective teacher–student relationships have been found to contribute to the engagement and academic outcomes of students (Engels et al., 2021). *Relational/rhetorical goal theory* explains that each student and teacher brings to the classroom their own expectations and experiences, and to have a successful learning process, instructors must meet the goals of students for being in the class: rhetorical or relational. Rhetorical goals focus on learning or task outcomes, and relational goals include perceived supportiveness, caring, and connectedness with others (Mottet et al., 2006). This theory explains that although rhetorical and relational goals could be considered independent, they are interrelated phenomena, as failing to achieve one goal could lead to failing to achieve the other goal. Recent studies provide evidence for this theory (Kaufmann and Frisby, 2017; Frisby et al., 2020). Finally, the *working alliance theory* conceptualizes teacher–student relationships as a collaborative working alliance. In this frame, the concept of working alliance in psychotherapy is applied to the classroom setting, emphasizing that the emotional bond between teacher and student and their collaboration in achieving the goals and tasks of their work together influence achievement (Toste et al., 2015). Noble et al. (2020) found that the ratings of the working alliance of students predicted their reports of risk of dropout mediated by school engagement.

Despite differences regarding the mechanisms for the effect of teacher–student relationships on engagement and achievement in the above-mentioned theories, important and consistent research findings stress the importance of teacher–student relationships in the experiences of high school students (Roorda et al., 2011, 2017; Quin, 2017).

However, again, the focus of most research about teacher–student relationships has been on students with TD, with less and inconclusive evidence about the effect of these relationships in SEN students (see Roorda et al., 2011). Thus, specific research in this regard is needed (Sabol and Pianta, 2012; Ewe, 2019), especially in inclusive settings (Pennington and Courtade, 2015) and considering their emotional, social, and/or learning difficulties (Murray and Greenberg, 2001; Murray and Pianta, 2007).

The research conducted on this topic indicates that SEN students have poorer teacher–student relationships than their typical developed peers (Murray and Greenberg, 2001; Al-Yagon and Mikulincer, 2004; Freire et al., 2020), and according to Henricsson and Rydell (2004), these relationships tend to be stable over time in elementary school for SEN students. In addition, most research on the teacher–student relationships of students with SEN is limited to the upper years of primary schools (for an exception, see Freire et al., 2020); thus, studying these relationships as the high school level is even more important.

This study analyzes the impact of teacher–student relationships and SEN on engagement and disengagement of students in a sample of SEN and TD secondary students in mainstream schools. Trying to fill the gaps in the literature on the engagement of SEN students, we used three widely agreed dimensions of engagement in this study: cognitive, emotional, and behavioral (Fredricks et al., 2004), with the addition of social engagement (Wang et al., 2017). Finally, we measure engagement and disengagement as separate continua.

## Method

### Design and Participants

This study used a non-experimental, correlational, and cross-sectional design to evaluate student engagement among adolescents with SEN and their TD peers. The inclusion criteria for the SEN group were (a) being enrolled in the 9th grade, (b) being in the inclusion program at a mainstream school, and (c) having a SEN diagnosis. For the TD group, the criteria were (a) being enrolled in the 9th grade, and (b) not having being diagnosed with SEN. The exclusion participation criterion for both groups was having autism (*n* = 16). Schools provided information regarding diagnoses to verify compliance with the inclusion criteria.

Participants were 9th-grade students recruited from 38 public mainstream schools from the Biobio Region in Chile. All schools were in urban areas and all enrolled SEN students as mandated by Chilean legislation. There were 340 students with SEN (306 with learning disabilities, 90% of the SEN group; 21 with attention deficit disorder, 6%; six with motor disability, 2%; four with a mild hearing impairment, 1%; and three with a mild visual impairment, 1%). Furthermore, 640 TD students participated in the study. The overall group included 575 female students (56%) and 445 male students (44%), with the gender breakdown being similar between groups [$\chi_{\left(1\right)}^{2}$ = 2.040; *p* = 0.153]. Note there was a slight age difference [*t*_(946)_ = 3.146; *p* = 0.002]. The mean age in the SEN group was 15.01 years (*SD* = 0.94) and 14.82 (*SD* = 0.86) for the TD group, that is, SEN students were on average 3 months older than TD students. Regarding economic status, 82.5% of the TD and 87.7% of the SEN sample had a family income below 690 USD, which corresponds to a low socioeconomic status.

### Instruments and Variables

(a) Special educational needs: The inclusion program for students with SEN to attend mainstream schools in Chile—called the school integration program—requires that students have a medical and psychological evaluation to identify their special need(s) prior to enrolment. The relevant Decree 170 (2009) states that SEN students enrolled in public mainstream schools to receive academic support from a special needs teacher along with attending regular classes. This is done in both the classroom and in a special resource room, allowing for more individualized assistance.

(b) The engagement measures of teacher–student relationships, peer support at school, and family support for learning were assessed with the subscales *teacher*–*student relationship* (nine items: “My teachers are there for me when I need them”), *peer support at school* (six items: “Other students at school care about me”), and *family support for learning* (four items: “My family/guardian(s) want me to keep trying when things are tough at school”) of the Student Engagement Inventory (SEI; Appleton et al., 2006). Although this instrument is called “student engagement,” the nature of its items better captures factors that influence engagement than indicators of student engagement *per se* (Veiga et al., 2014). Each item was answered on a four-point Likert scale (1 = strongly disagree, 2 = disagree, 3 = agree, and 4 = strongly agree) as in the original instrument (Appleton et al., 2006). The omission of the midpoint in a Likert scale to measure attitude is debated. However, we decided not to change the number of options because that could alter the psychometric properties of the instrument. Furthermore, omitting a neutral option could in some circumstances be beneficial in terms of forcing the respondent to choose an answer in areas with high social desirability pressures (Chyung et al., 2017), which could be the case in this study.

Reliability indices in the Chilean validation process were between ω = 0.76 and ω = 0.88 for all scales. The reliability indices in the present sample were ω = 0.875, ω = 0.785, and ω = 0.700 for teacher–student relationships, peer support at school, and family support for learning subscales, respectively. In the validation sample, the SEI showed a good fit for the proposed six-factor model (Appleton et al., 2006), and its factorial invariance has been demonstrated in various countries (Virtanen et al., 2017) including Chile (Espinoza et al., 2018).

(c) The perception of school participation was measured with the subscale *positive perception of school participation* (six items: “At my school, all students have the chance to participate”) from the School Participation Scale developed by John-Akinola and Nic-Gabhainn (2014). This subscale measures if students perceive that school participation is real or symbolic in their educational institution. Each item is answered on a five-point Likert scale (1 = strongly disagree; 5 = strongly agree). This study used the Spanish version, which has been validated in a sample of 1,428 students in secondary schools in central-southern Chile (*M* = 15.59; *SD* = 1.52) (Pérez-Salas et al., 2019). Reliability in the Chilean validation process was ω = 0.877 for this subscale (Pérez-Salas et al., 2019), and in the present sample, it was α = 0.857 (ω = 0.868).

(d) Engagement and disengagement were measured with the Multidimensional Scale of School Engagement (MSSE; Wang et al., 2017). It consists of 37 items that assess engagement and disengagement on a five-point Likert scale. The engagement factor contains 19 items: (a) *behavioral engagement* (four items: “I ask questions when I don't understand”), (b) *cognitive engagement* (five items: “I look over my schoolwork and make sure it is done well”), (c) *emotional engagement* (five items: “I am happy at school”), and (d) *social engagement* (five items: “I enjoy working with peers at school”). The disengagement factor contains 18 items: (a) *behavioral disengagement* (eight items: “I don't follow school rules”), (b) *cognitive disengagement* (two items: “Finishing my homework fast is more important to me than doing it well”), (c) *emotional disengagement* (four items: “I feel overwhelmed by my schoolwork”), and (d) *social disengagement* (four items: “I don't care about the people at my school”). This instrument was validated by the Pérez-Salas (2021) among Chilean students. The reliability indices in the present sample for the engagement factor were α = 0.902 (ω = 0.902) and α = 0.869 (ω = 0.869) for the disengagement factor. These indices were similar to those found in the validation process in Chile (Pérez-Salas, 2021).

### Procedure

This study is part of ongoing longitudinal research on engagement trajectories of high school students. The data for this particular study is from the first wave of data collection, and the experiment was conducted during the second semester of the school year (August/December 2018). The ethical committee of the Universidad de Concepción of the First Author approved this research, and both the school boards of each city and the school gave their authorization. After this, eligible participants were determined according to the study inclusion criteria for both samples (students with SEN or students who were TD).

An invitation to participate in the study was sent to the parents of eligible participants. After explaining the rights and the purpose of the study of students and obtaining active informed consent from the parents and student informed assent, trained psychologists gave the instruments to TD students for self-administration, and individually assessed SEN students using a reading aloud application format. We decided to use different methods because difficulties in applying self-administration questionnaires in SEN students have been identified (Finlay and Lyons, 2001; Goegan et al., 2018), suggesting that accommodations should be made (Goegan et al., 2018). However, to ensure there was not a skew from the application format, we conducted a quasi-experimental study with another sample that showed that the application format (self-administered vs. read aloud) had no effect, confirming similar reliability indexes for both samples^1^.

The evaluations were conducted in schools of participants and lasted approximately 45 min. Participants received a movie ticket for their collaboration.

### Data Analysis

The percentage of missing data was evaluated by item and participants, and then missing values were replaced with the Expectation-Maximization imputation method to enable analysis with all cases.

As the SEN participants had different conditions (learning, sensorial, and motor disabilities), we analyzed if there were differences in their engagement and disengagement before conducting the main analysis. Furthermore, before the analysis, we tested compliance with the assumptions of the parametric technique: normal distribution with asymmetry and kurtosis, and the homogeneity of variances with a Box's M test and Levene's test. Heteroscedasticity corrections were made when needed. Finally, to evaluate possible differences between groups (SEN vs. TD), we performed a multivariate analysis of variance with engagement and disengagement dimensions. We employed SPSS, version 25 (IBM, 2017) for all the analyses.

## Results

The total missing values per item in the sample were <1% across cases. We had full data for 80.8% of the participants (81 items) and only four individuals (0.4% of the sample) had omitted 6–18 items (7–22%) in their protocols. As mentioned, missing data were replaced with the Expectation-Maximization imputation method to enable the analysis with all available data (*N* = 1,020). A multivariate ANOVA did not indicate differences between participants with different SEN conditions when it came to student engagement [*F*_(16, 1340)_ = 0.909; *p* = 0.558; $\eta_{\text{p}}^{2}$ = 0.011] or disengagement [*F*_(16, 1340)_ = 0.645; *p* = 0.849; $\eta_{\text{p}}^{2}$ = 0.008]. Thus, we decided to treat all SEN participants as one group. Asymmetry and kurtosis values were lower than I2I in all dependent variables in both samples, supporting compliance of the assumption of the normal distribution of the variables.

Table 1 shows the mean, SD, *t-*tests, and effect sizes for teacher–student relationships, peer support at school, family support for learning, and perception of school participation for students with SEN and TD. Results indicate good levels of teacher–student relationships, peer support at school, and family support for learning, and very positive perceptions of school participation in both TD and SEN students. Mean comparisons revealed that SEN students report having better teacher–student relationships and an even more positive perception of school participation than do TD students. No group differences were found in peer support at school or in family support for learning.

**Table 1:** Mean, SD, *t*-tests, and effect sizes for teacher–student relationship, peer support at school, family support for learning and perception of school participation in students with SEN and TD.

**Variable**	**Students with SEN (*****n*** **=** **340)**		**Students with TD (*****n*** **=** **680)**		**Group comparisons**	
	***M***	***SD***	***M***	***SD***	***t*_**(df)**_**	**$\eta_{\text{p}}^{2}$**
Teacher–student relationships	3.097	0.534	2.792	0.585	*t*_(735.23)_ = 8.323^**^	0.06
Peer support at school	3.100	0.540	3.043	0.551	*t*_(1018)_ = 1.556	
Family support for learning	3.461	0.600	3.433	0.560	*t*_(1018)_ = 0.724	
Perception of school participation	4.107	0.786	3.771	0.855	*t*_(1018)_ = 6.068^**^	0.035
**Student engagement**						
Behavioral engagement	3.511	0.809	3.487	0.816	*F*_(1, 1018)_ = 0.199	
Cognitive engagement	3.728	0.800	3.568	0.864	*F*_(1, 1018)_ = 8.145^**^	0.008
Emotional engagement	4.064	0.751	3.744	0.832	*F*_(1, 1018)_ = 35.628^**^	0.034
Social engagement	3.996	0.754	3.751	0.845	*F*_(1, 1018)_ = 20.480^**^	0.020
**Student disengagement**						
Behavioral disengagement	2.504	0.871	2.270	0.793	*F*_(1, 1018)_ = 18.383^**^	0.018
Cognitive disengagement	2.551	1.084	2.243	1.018	*F*_(1, 1018)_ = 19.945^**^	0.19
Emotional disengagement	2.555	0.955	2.580	0.956	*F*_(1, 1018)_ = 0.160	
Social disengagement	1.959	0.913	2.026	0.927	*F*_(1, 1018)_ = 1.183	

*SEN, special educational needs; TD, typical development*.

** *p < 0.01*.

The multivariate ANOVA showed a significant statistical difference between SEN and TD students for the student engagement indicators (behavioral, cognitive, emotional, and social) [*F*_(4, 1015)_ = 12.484; *p* < 0.001; $\eta_{\text{p}}^{2}$ = 0.047], although both had good levels (Table 1). The intersubjects effect test showed that cognitive, emotional, and social engagement were higher in SEN students than TD students (*p* < 0.01) (Table 1). This means that students with SEN reported working harder at school, having more fun at school, and enjoying spending time with their peers at school more than those with TD.

Regarding the student disengagement dimension, a significant statistical difference was found between SEN and TD students in the indicators (behavioral, cognitive, emotional, and social) [*F*_(4, 1015)_ = 10.173; *p* < 0.001; $\eta_{\text{p}}^{2}$ = 0.039]. In general, SEN and TD students had low levels of behavioral, cognitive, and emotional disengagement, but both groups reported some degree of social disengagement. The intersubjects effect test showed that cognitive and behavioral disengagement were higher in SEN students than TD students (*p* < 0.01) (Table 1). This means that students with SEN reported more maladaptive behaviors at school and more disaffection with learning than their peers with TD (*p* < 0.01). No differences were found between samples in emotional disengagement or social disengagement.

Next, using the stepwise method, linear regressions were analyzed to predict the scores in each engagement and disengagement dimension for teacher–student relationships, peer support at school, family support for learning, and perception of school participation.

For behavioral engagement, the regression model included perceptions of school participation, peer support at school, teacher–student relationships, and group (SEN vs. TD) was statistically significant [$R_{\text{adj}}^{2}$ = 0.232, *F*_(4, 1015)_ = 77.991, *p* < 0.001]. Of the predictive variables, the most important was the positive perception of school participation, followed by peer support at school, teacher–student relationships, and group (TD). That is, the better the perceptions of (a) school participation opportunities, (b) peer support, and (c) teacher–student relationships, along with (d) being TD, the higher the scores for behavioral engagement, in that order (Table 2).

**Table 2:** Linear regression models for behavioral, cognitive, emotional, and social engagement dimensions.

**Dependent variable**	**Model**	**B**	**SE**	**β**	***t***
Behavioral engagement	(Constant)	1.195	0.144		8.304^***^
	Perception of school participation	0.053	0.005	0.334	9.872^***^
	Peer support at school	0.029	0.008	0.119	3.723^***^
	Teacher–student relationships	0.022	0.006	0.141	3.897^***^
	Group (SEN/TD)	−0.153	0.049	−0.089	−3.117^**^
Cognitive engagement	(Constant)	1.382	0.163		8.466^***^
	Perception of school participation	0.046	0.006	0.275	8.047^***^
	Teacher–student relationships	0.026	0.006	0.160	4.303^***^
	Family support for learning	0.036	0.012	0.099	3.074^**^
Emotional engagement	(Constant)	0.537	0.119		4.496
	Perception of school participation	0.070	0.004	0.436	15.666^***^
	Teacher–student relationships	0.038	0.005	0.245	8.359^***^
	Peer support at school	0.037	0.007	0.150	5.716^***^
Social engagement	(Constant)	0.550	0.127		4.328^***^
	Perception of school participation	0.062	0.005	0.381	12.959^***^
	Peer support at school	0.083	0.007	0.332	11.985^***^
	Teacher–student relationships	0.012	0.005	0.077	2.483^**^

*SEN, special educational needs; TD, typical development*.

** *p < 0.01*;

*** *p < 0.001*.

For cognitive engagement, the regression model included teacher–student relationships, family support for learning, and perception of school participation [$R_{\text{adj}}^{2}$ = 0.190, *F*_(3, 1016)_ = 80.656; *p* < 0.001]. Among the predictive variables, the most important was again positive perception of school participation, followed by teacher–student relationships and family support for learning (Table 2). This model implies that the better is (a) perception of school participation opportunities, (b) teacher–student relationships, and (c) family support for learning, the higher are the scores for cognitive engagement.

For emotional engagement, the regression model was statistically significant and included perception of school participation, teacher–student relationships, and peer support at school [$R_{\text{adj}}^{2}$ = 0.477, *F*_(3, 1016)_ = 311.276, *p* < 0.001]. Among the predictive variables, the most important was positive perception of school participation, followed by teacher–student relationships and peer support at school (Table 2). This model implies that the better is (a) perception of school participation opportunities, (b) teacher–student relationships, and (c) peer support at school, the higher are the scores for emotional engagement.

For social engagement, the regression model that included perception of school participation, peer support at school, and teacher–student relationships was statistically significant [$R_{\text{adj}}^{2}$ = 0.415, *F*_(3, 1016)_ = 242.155; *p* < 0.001]. Among the predictive variables, the most important was again positive perception of school participation, followed by peer support at school and teacher–student relationships (Table 2). This model implies that the better is (a) perception of school participation opportunities, (b) peer support at school, and (c) teacher–student relationships, the higher are the scores for social engagement.

For behavioral disengagement, the regression model that included all predictive variables was statistically significant [$R_{\text{adj}}^{2}$ = 076, *F*_(5, 1014)_ = 17.728; *p* < 0.001]. Among the predictive variables, the most important was group (SEN = 1), followed by teacher–student relationships (–), peer support at school (+), perception of school participation (–), and family support for learning (–) (Table 3). This model means that (a) having SEN, (b) having poorer teacher–student relationships, (c) higher peer support at school, (d) perception of scarce opportunities to participate at school, and (e) lower support from families for learning leads to higher scores for behavioral disengagement.

**Table 3:** Linear regression models for behavioral, cognitive, emotional, and social disengagement dimensions.

**Dependent variable**	**Model**	**B**	**SE**	**β**	***t***
Behavioral disengagement	(Constant)	3.251	0.180		18.044^***^
	Teacher–student relationships	−0.023	0.007	−0.150	−3.575^***^
	Group (SEN/TD)	0.330	0.055	0.188	5.994^***^
	Perception of school participation	−0.019	0.006	−0.117	−3.160^**^
	Peer support at school	0.032	0.009	0.128	3.533^***^
	Family support and learning	−0.040	0.013	−0.111	−3.093^**^
Cognitive disengagement	(Constant)	3.667	0.215		17.030^***^
	Perception of school participation	−0.036	0.007	−0.176	−5.456^***^
	Group (SEN/TD)	0.387	0.069	0.174	5.629^***^
	Family support and learning	−0.044	0.015	−0.096	−3.017^**^
Emotional disengagement	(Constant)	4.243	0.156		27.117^***^
	Perception of school participation	−0.036	0.007	−0.191	−5.300^***^
	Teacher–student relationships	−0.034	0.007	−0.187	−5.115^***^
	Group (SEN/TD)	0.140	0.062	0.069	2.252^*^
Social disengagement	(Constant)	4.288	0.166		25.837^***^
	Peer support at school	−0.077	0.009	−0.275	−8.833^***^
	Perception of school participation	−0.037	0.006	−0.205	−6.578^***^

*SEN, special educational needs; TD, typical development*.

* *p < 0.05*;

** *p < 0.01*,

*** *p < 0.001*.

For cognitive disengagement, the regression model including the positive perception of school participation, having SEN, and family support for learning was statistically significant [$R_{\text{adj}}^{2}$ = 0.065, *F*_(3, 1016)_ = 26.67, *p* < 0.001]. Among the predictive variables, the most important was the perception of school participation (–), followed by group (SEN = 1) and family support for learning (–) (Table 3). This model implies that (a) having SEN and (b) a poorer perception of both school participation opportunities, and (c) family support for learning will lead to lower scores for cognitive disengagement.

For emotional disengagement, the regression model including the positive perception of school participation, teacher–student relationships, and the group was statistically significant [$R_{\text{adj}}^{2}$ = 0.103, *F*_(3, 1016)_ = 39.943, *p* < 0.001]. Among the predictive variables, the most important was the perception of school participation (–), followed by teacher–student relationships (–) and group (SEN = 1) (Table 3). This model suggests that (a) having SEN and (b) a poorer perception of school participation opportunities, and (c) a negative perception of teacher–student relationships will lead to lower scores for emotional disengagement.

Finally, for social disengagement, the regression model including peer support at school and perception of school participation was statistically significant [$R_{\text{adj}}^{2}$ = 0.159, *F*_(2, 1017)_ = 97.48, *p* < 0.001]. Among the predictive variables, the most important was peer support at school (–), followed by the perception of school participation (–) (Table 3). This model means that (a) the poorer the perception of peer support at school and (b) a negative perception of school participation opportunities leads to lower scores for social disengagement.

## Discussion

Few studies have measured the engagement and disengagement of students with SEN, and even fewer have examined the impact of factors such as teacher–student relationships on their engagement and disengagement in school. This cross-sectional study extended prior research investigating student engagement in a sample of SEN and TD students measuring this construct in a multidimensional manner (cognitive, behavioral, emotional, and social), while considering engagement and disengagement as separate but related phenomena.

Inconsistent with previous research, we found engagement of SEN students was higher than that of TD students for the cognitive, emotional, and social indicators. We also found no differences between both groups for the behavioral indicator. Much of the literature in this field suggests that SEN students could be conceptualized as at risk for low engagement due to their struggles at school (Douglas et al., 2012; Cortiella and Horowitz, 2014; Moreira et al., 2015). In addition, previous research reported lower levels of engagement in this population than in TD students (Lovelace et al., 2014; Schindler, 2018).

Scant research has directly examined the construct of cognitive engagement for students with SEN (O'Donnell and Reschly, 2020). However, O'Donnell and Reschly (2020) highlight that academic difficulties experienced by students with SEN may reflect a lack of self-regulation strategies and thus could impact engagement. Our results contradict this, showing that SEN students present higher scores in cognitive engagement. According to the conceptualization of cognitive engagement in the MSSE used in this study, this finding means that the SEN students in our sample reported “higher metacognitive strategies (…) to productively coordinate their energy and behavior in school” (Wang et al., 2017, p. 12). This contradictory finding is explainable because multiple studies have shown that students with SEN can successfully learn metacognitive skills [for more detail, see the meta-analysis of de Boer et al. (2018) and Donker et al. (2014)]; thus, the work of special needs teachers with SEN students at schools could be reflecting the positive results thereof in their higher scores for cognitive engagement in our study.

Our findings also contradict previous research reporting that emotional engagement in students with SEN is lower than that in TD students. This could be attributed to the different conceptualization of emotional engagement in various studies. In the MSSE, Wang et al. (2017, p. 3) state that emotional engagement represents “the external manifestations of students' feelings regarding school” (having fun at school, being happy at school, being proud of their school, and being interested in what they are learning at school) and do not include facilitators of engagement (contextual predictors). Our results also show higher social engagement scores for SEN students than for the TD group, reflecting the very good quality of this involvement of adolescents in social interactions (enjoy working with peers at school, enjoy spending time with peers at school, and openness to working with peers and making friends at school). These results are encouraging for SEN education, since the importance of positive emotions for development and well-being has been emphasized by positive psychology (Norrish and Vella-Brodrick, 2009; McKeering et al., 2021).

Aligned with the self-system model theory (Connell and Wellborn, 1991) and our hypothesis, our findings show that close relationships with teachers positively contribute to all dimensions of student engagement in our sample, an effect consistently reported in research in this field with TD students (Roorda et al., 2011, 2017; Quin, 2017). We also found that the higher was the perception of opportunities to participate at school, the higher were all indicators of engagement (cognitive, emotional, behavioral, and social), reflecting the relevance of school-level and classroom-level variables in student engagement (Fredricks et al., 2004).

However, the better teacher–student relationships and more opportunities to participate at school reported by SEN students compared to their TD peers were unexpected findings of our study. Previous research mostly reported poorer relationships between SEN students and their teachers (Murray and Greenberg, 2001; Al-Yagon and Mikulincer, 2004; Murray et al., 2006; Freire et al., 2020) and fewer opportunities to participate at school than TD students (Coster et al., 2013). Our results show the opposite, as Schwab and Rossmann (2020) similarly showed in a recent study that found SEN students rated their teacher–student relationships more positively than TD students.

O'Donnell and Reschly (2020) state that the inconsistence in school connectedness or teacher–student relationships in the literature on SEN students could be attributable to the availability of resource rooms and close relationships with special education teachers in each context. Similarly, Schwab and Rossmann (2020) explain their results by arguing that in the Austrian school system, SEN students are often supported by two teachers in regular classrooms, one of whom is a special needs teacher who spends much time with the students, providing opportunities to develop a closer relationship with them. We think the same hypothesis could explain our positive results for teacher–student relationships and the better perception of participation of the SEN students in our sample, as such students attending public mainstream schools in Chile receive academic support by special needs teachers in regular classrooms and additional support in small groups in a special resource room. This reflects the increased time special needs teachers spend with these students and that these teachers may be more sensitive to their needs.

The positive effect of special needs teachers for Chilean students is also supported by a qualitative study that we conducted with a sample of adolescents with learning disabilities^2^. Based on the perceptions of students, that study concluded that special needs teachers are crucial for their engagement, as their pedagogical practices are oriented to satisfy the needs of students for competence and relatedness, aspects that have been shown as key in adjusting to school (Connell and Wellborn, 1991).

Exposure to more positive relationships with special needs teachers could also explain the better teacher–student relationships reported by SEN students and their higher emotional and social engagement scores. This is aligned with the study of Martin and Collie (2019) that predicted greater engagement of high school students as to when the number of positive relationships outnumbered negative relationships with their teachers.

Finally, we found significant statistical differences between SEN and TD students for some disengagement indicators. On the one hand, engagement of SEN students was higher in the cognitive, emotional, and social dimensions; however, on the other hand, they also had higher scores for cognitive and behavioral disengagement. These results emphasize that engagement and disengagement are two distinctive phenomena (Skinner et al., 2009). Thus, although students with SEN report working harder at school, enjoying being at school and studying, and have positive interactions with others at school, they also perceive higher “disaffection” with learning (Skinner et al., 2008) than their TD peers. This should alert educators, as it could lead SEN students to gradually withdraw from the social environment in response to negative experiences (Finn, 1989).

A possible explanation for this apparent contra-intuitive result for SEN students (high cognitive and behavioral disengagement alongside high cognitive and emotional engagement) might be because according to a meta-analysis, the relationship between academic achievement and engagement is not always conclusive (Lei et al., 2018). Therefore, although extensive empirical research on the relationship between academic achievement and engagement exists, some scholars have found non-significant associations between these variables (Lei et al., 2018). Possibly, this is because students who achieve good grades better master the abilities needed for easier learning than low achievement students, and so apply less effort and strategies when studying (Lei et al., 2018). We think this hypothesis could be applicable to our results, meaning the better cognitive engagement of SEN students may reflect their extra educational effort compared to their classmates. Furthermore, despite that they seem to enjoy being at school, being with peers, and learning, they may be starting to experience a higher level of cognitive and behavioral disengagement, perhaps because they feel some frustration when learning.

### Implications for Policies and Educational Practice

The current study provides evidence of the need for continuing research on students with SEN to unpack the conditions that provide support or hinder their participation and achievement in schools. Overall, this research suggests that teachers have a relevant influence in all dimensions of engagement of students and on emotional and behavioral disengagement for TD and SEN students. At the same time, the positive relationship between teachers and students was inversely associated with the disengagement of students. These findings are particularly relevant for students with SEN who often experience more struggles in school and higher dropout rates.

These results have implications for policy and practice. We hope this study will inform policymakers and authorities when drafting policies regarding students with SEN, especially when it comes to the relevance of teacher–student relationships in the achievement and well-being of students. In addition, this study highlights the relevance of including students with SEN in research. Authorities must consider this when evaluating topics impacting the trajectories of students.

Regarding implications for practice, it would benefit school systems to structure student interactions in ways to strengthen opportunities to provide academic and emotional support. School districts and administrators have an important role in providing professional development to improve the abilities of teachers to create strong teacher–student relationships. In the case of inclusive education, students with SEN have the additional support of special education teachers, which could impact their perceptions of teacher–student relationships, as the additional support could provide further opportunities to enhance these relationships. Schools should also make efforts to ensure that both TD and SEN students feel like there are plenty of opportunities to engage in school participation, since that was also a key factor.

### Limitations and Future Research

Despite the strengths of this study, some limitations must be considered when interpreting its results. First, this is a correlational and cross-sectional study; thus, no cause–effect conclusions should be derived from our results. Second, all our measures rely on self-reporting of students. It would have been informative to have impressions of teachers on teacher–student relationships and more direct measures of school participation opportunities to disentangle in terms of whether the level of opportunities, belief that there are many opportunities or a combination of both have an impact.

Future quantitative work should examine practices of teachers to help determine what creates good teacher–student relationships, and what other impacts teachers may have on engagement and disengagement dimensions of students (cognitive, behavioral, emotional, and social). Furthermore, qualitative work (e.g., interviews with teachers, and TD and SEN students) should be considered to provide detailed insight into how such relationships are created and if specific factors have a greater influence on the performance and well-being of students. In this regard, mixed research methods could be a productive approach to collect comprehensive data to better understand the experiences of students, particularly those who face barriers to participating in schools.

Despite our limitations, this study adds to a fairly limited field of research. It includes a relatively large sample of students with SEN studying in mainstream settings, whom it compared with their TD peers. Simply focusing on the engagement and disengagement of students with SEN is a contribution to this field considering the lack of information on both constructs for this more vulnerable population. It also suggests clear future paths for additional research and potential school-level improvements.

Finally, we hope this article draws attention to the challenges faced by SEN students and the relevance of teacher–student relationships in contributing to both engagement and disengagement depending on the quality of these relationships. These findings suggest clear future paths for additional research and potential school-level interventions to strengthen student engagement and avoid the negative consequences of disengagement.

## Data Availability Statement

The raw data supporting the conclusions of this article will be made available by the authors, without undue reservation.

## Ethics Statement

The studies involving human participants were reviewed and approved by Comité de Ética, Bioética y Bioseguridad de la Vicerrectoría de Investigación y Desarrollo de la Universidad de Concepción, Concepción, Chile. Written informed consent to participate in this study was provided by the participants' legal guardian/next of kin.

## Author Contributions

All authors contributed to this manuscript by doing secondary research, conceptualizing the methodology, and drafting and revising the manuscript.

## Conflict of Interest

The authors declare that the research was conducted in the absence of any commercial or financial relationships that could be construed as a potential conflict of interest.

## Footnotes

^1^Pérez-Salas, C. P., Parra, V., Sáez, F., Ramírez, P., and Zañartu, I. (2021). Self-administered versus read aloud questionnaires: Quasi-experimental study in students with and without learning disabilities.

^2^Lara, G., González, N., Lara, F., Lagos, L., Parra, V., and Pérez-Salas, C. P. (2021). Relación docente-estudiante y compromiso escolar: percepción de jóvenes con necesidades educativas especiales (Manuscript submitted for publication). Departamento de Psicología, Universidad de Concepciín.
